# Rationalism in Medicine

**Published:** 1874-01-15

**Authors:** J. F. Todd

**Affiliations:** Kewanee, Ill.


### RATIONALISM IN MEDICINE.

An Essay Read Before the Military Tract Medical Association, by J. F. Todd, M.D., of Kewanee, Ill.

THE signification of the term rationalism, must of necessity, correspond to the mental development of the human race. A medical theory which was accepted in good faith in the last century, and celebrated by the good Queen Anne by the imposition of royal hands upon the celebrated Dr. Johnson, is received with derision and ridicule in this century. The belief in the royal touch and the royal prerogative have passed away. The tendency of modern thought and investigation is toward a rigid analysis of supposed facts, musty traditions, irrational theories, and false dogmas, tried in this crucible, and regarded with as little concern by these chiefs of rationalism as though they had never played an important part in any of the concerns of life. There has ever been, and probably there will ever be, earnest disputes between rival schools in the arts and sciences, each claiming to represent the most rational ideas, and to dispense the greatest practical benefits.

The term rationalism may be appropriated by any sect, to conceal the extravagances of any dogma. In ancient times, all of the mysteries of nature were attributed to the agency of supernatural causes; and disease in its varied forms was supposed to result from the rage of some offended god. The rules of practice were as irrational as the theories which gave them birth; and yet, both theory and practice were received in good faith; the first as rational theory, the latter as practical art. The early history of medicine exposes a tedious series of mistakes and absurdities, resulting from vain attempts to base science upon theories. And when we reflect that the origin of earthquakes was attributed to demoniacal rage, and every natural phenomenon to the exercise of supernatural powers, we confessedly claim the science of medicine represented the rationalism of the age in which it existed. Profoundly ignorant of the anatomy and physiology of the human body, they occupied their time with speculations as to the mode of generation, and the habitation of the soul.

It is not within the scope of this paper to give even a resume of the various sects of medical philosophers who pretended to teach correct views of pathology. It is sufficient for the purpose to remind you that all that we can learn of ancient medical theory gives us but an imperfect idea of confused and absurd dogmas. The earliest emergence from this motley assemblage of facts and false hypotheses, results from the teachings of Hippocrates; and from his time we date the beginning of the development of rational medical practices. Too wise to accept the prevalent dogmas, and too sincere to accept canonization after the fashion of his predecessors, he sought a guide for study and a rule of practice which we can safely and loyally adopt in this 19th century, and the correction of all deviations from the natural condition of the human body by methods of treatment founded upon attentive observation. He advised as to the investigation of diseased action, the medical treatment, and the regimen, in a manner which justly entitles him to the respect of every votary of medical science.

It is true, that his rules of practice betray the general ignorance which then prevailed of physiological laws; but his aphorisms denote a happy perception of an important fact, one which future ages will always appreciate. In order to correct unnatural conditions, we must understand the natural condition; and we must remember the existence of the grand conservative principle of life. Hippocrates ranks among the first and best of the early disciples of medicine to accord to natural laws their pre-eminent value in the correction of unnatural conditions, and the first to insist upon the careful study of nature. In order to fully appreciate the value of his services, we must remember that anatomy, chemistry, and physiology, were then almost unknown, and that his rules of practice were mainly deduced from intelligent empiricism, and applied with a worthy zeal for the benefit of his fellow-men. It must be admitted that frequent and wide departures from the course of study advised by him were soon observed; and medical teachers, anxious to attract attention, relapsed into the old methods of speculation; but so profound had been the impression produced by the doctrines of Hippocrates, that the only safe and direct road to success was at last found in the admission of his title to leadership. The history of medicine (the history of civilization) from that time until the present, has chronicled the same successes and reverses, triumphs and defeats. Under priestcraft and kingcraft, and the mental serfdom and physical servitude of the middle ages, scientific progress was delayed. The superstitious horror of dissecting the human body, and the monopoly of medical practice by an arrogant priesthood, effectually barred the paths of knowledge. Under the influence of the general awakening to a spirit of inquiry, under the teachings of Luther, the study and practice of medicine received a new and healthy impulse. Previous to that time, the approving and licensing practitioners had been committed to the bishops, and medical practice was accordingly engrossed by ignorant monks. At this juncture, the discoveries in anatomy were so remarkable as to distinguish the rational method of observation, and to rebuke the presumptuous pretensions of ignorant empiricism. Vesalius exposed the errors of the Galenian system with as complete success as did Luther the fallacies of the Romish Church. At this time, Linacre founded the College of Physicians and Surgeons, in London; and Fabricius made startling discoveries in anatomy, and instructed the immortal Harvey in the truths of medical science. I can pay no fitting tribute to Harvey's great discovery; it needs none; its praise is recorded in nearly every page of printed matter uttered from the press, and its benefits are enjoyed by the entire human race. Next in importance, comes the discovery of vaccination, which has robbed a fearful scourge of its greatest terrors, and preserved the lives of thousands of our fellow-creatures. With this brief retrospect of the history of medicine, we are bound to assume that medical science is not only rational but progressive, and that it challenges our admiration and loyal support; that medical art is necessary and beneficent, and deserves at our hands a complete vindication of its claims to general respect and confidence. The most *intelligent* of the reading public have ignored its claims. They ask us for cures for real or imaginary maladies; and we dispense drugs of real potency, and whether for good or evil, depends upon our sagacity, or upon some hidden influences which no ordinary human skill can detect. Whatever of success we have achieved, must be accepted as a measure of the intelligence and zeal of the disciples of our art; and while we understand, far better than the public, the uncertainties attending our efforts, and the discouragements incidental to the great responsibilities we are bound to assume, we also better appreciate the triumphs of rational medicine. The history of medical science reflects the prevailing philosophies of every age; and its improvement has kept even pace with the mental development of the people. History is its sufficient vindication.

We found our claim to the term rationalism, not only upon the valuable results already achieved, but also upon the experience of our daily practice. We treat disease as a preternatural physiological process; and while we are forced to admit that medical science is the science of probabilities, we are assured by our own observation, and the testimony of our patients, that our efforts in their behalf are often completely successful. *With the assistance of nature, we cure disease.* Consistent faith is necessary to successful effort, or profitable study. Nothing has tended more to degrade our calling than the disparagement of medicine by disappointed physicians, who hastily reject rich grains of truth in ridding themselves of the light husks of falsehood. It is due to our profession to labor for its improvement, and to increase its usefulness. The rational practitioner must be a student of nature, as well as a studious reader. He must possess the quality of intellect which will lift him above the plane of partisanship, and the scholarship necessary to enable him to apply scientific facts, and understand natural phenomena. He must identify himself with the various movements of the age having for their object the improvement of the race, that his mission will be so well performed that his influence will live after he is gone.

